# Study on the characteristics of head and neck movements of geese walking in a straight line at different speeds

**DOI:** 10.3389/fvets.2025.1554240

**Published:** 2025-05-30

**Authors:** Jiajia Wang, Zheng Zhang, Xinming Jiang, Dongyan Huang, Wei Song, Fu Zhang, Zhihui Qian, Lei Ren, Luquan Ren

**Affiliations:** ^1^College of Engineering and Technology, Jilin Agricultural University, Changchun, China; ^2^College of Agricultural Equipment Engineering, Henan University of Science and Technology, Luoyang, China; ^3^Key Laboratory of Bionic Engineering, Ministry of Education, Jilin University, Changchun, China

**Keywords:** motion analysis, bird neck, goose, walking, head bobbing

## Abstract

**Background:**

The avian cervical spine, a crucial anatomical structure connecting the cephalic and thoracic regions, serves a critical function in maintaining visual stability during locomotion. Extensive studies have documented characteristic head-bobbing behavior in small-bodied avian species (e.g., *Columba livia domestica* and *Coturnix coturnix*) during terrestrial movement. However, the kinematic patterns of *Anser anser domesticus* during ambulation across varying velocity parameters remain unexplored in current literature.

**Objective:**

To investigate whether head bobbing occurs during head and neck movements; to analyze differences between head and neck movement characteristics under different walking speeds.

**Methods:**

High-speed photography equipment was used to obtain images of domestic geese in different linear walking states (slow walking, normal walking, and fast walking) and to analyze gait changes and head-neck movement characteristics under varying movement states.

**Result:**

The results demonstrate that domestic geese exhibit nodding behavior similar to other birds, comprising thrust and hold phases. During a full nodding cycle, the thrust phase lasted significantly longer than the hold phase. Furthermore, the closer a goose's neck joint is to the trunk, the greater the joint angle variation observed across three motion states. This indicates that neck nodding depends on lower cervical joint motion. Concurrent analysis of head trajectories revealed horizontal stability during locomotion, with vertical fluctuation amplitude progressively decreasing as movement speed increased.

## 1 Introduction

Birds exhibit a variety of typical behaviors in their daily activities, among which two are particularly striking. One is the periodic back-and-forth swing of the head during walking, known as the “head-bobbing” movement (1–5). This movement pattern consists of two main stages: the hold phase and the thrust phase (6). In the hold phase, the bird's head remains stationary relative to the surrounding environment, while the body continues to move forward; in the thrust phase, the head moves rapidly forward, synchronizing with the body's movement (7). This unique movement mechanism helps birds to maintain visual stability while walking, reducing retinal image blur due to body movement (8, 9). The other is the gait characteristics of birds when walking (10, 11). For most terrestrial birds, walking is their preferred mode of locomotion at low speeds, and they may choose to run or jump for faster movement when needed. However, not all birds exhibit consistency in the transition from walking to running, with some small birds preferring to run even at moderate speeds rather than walk (12). van Bijlert et al. (13) resolved the paradox of birds' “ground-hugging gallop” through predictive gait simulations of emus (*Dromaius novaehollandiae*), demonstrating that from an energetic and muscular activation perspective, ground-hugging gallop is the optimal gait for birds. Furthermore, birds do not exhibit distinct gait changes like humans when transitioning from walking to running, but rather show a smooth transition (14, 15).

Ortega's research on “head swing” showed that visual cues are the main factors triggering head swing in birds, and this movement is restricted by the need for image stability and depth perception (16). According to the conclusions of Theunissen's experiments (17), in the face of different types of motor interference, pigeons' head stabilization mechanism depends on the feedback of multiple sensory systems, in which visual feedback plays a key role in some cases (e.g., translational motion). Besides, Fujita compared the walking patterns of blackhead gulls under different environmental and behavioral conditions to demonstrate that head swing affects not only visual perception but also significantly affects avian gait (18). Specifically, head swing can optimize visual input by adjusting gait parameters, allowing birds to more efficiently acquire environmental information during walking.

Previous studies have shown a strong association between walking gait and head movement in birds (19–21). The head swing is usually accompanied by a specific gait pattern and exhibits different features in different species of bird (22, 23). However, a recent study by Kumar (24) proposed a thought-provoking view of that a flexible neck expands the bird' visual field, resulting in a slower stride speed. This implies that the neck flexibility may be one of the key factors affecting gait change in birds.

Domestic geese are widely distributed and easily accessible, and the number of cervical vertebrae is usually between 17 and 18. These cervical vertebrae are tightly connected through the joints and form S-shaped curvature, providing excellent flexibility and stability to the geese neck. Wang et al. (25) obtained the structural parameters of the cervical spine of geese and ducks by using computed tomography technology and three-dimensional (3D) model reconstruction method. In addition, they also used the biplane X-ray dynamic motion capture system to directly obtain the intervertebral angular displacement of geese cervical bones, and analyze the three-dimensional dynamic motion of geese (26). In addition, they also used the high-speed photography technology to investigate the motion of goose neck in the narrow space, and use the method of magnetic resonance imaging to obtain the goose neck muscle anatomy (27). However, current studies on domestic goose necks focus on their anatomical features and stabilization mechanisms. It remains to be revealed whether the goose has this typical “nodding” movement when walking, and no reports are the difference between the “nodding” movement at different movement speeds.

Therefore, the main purpose of this study including two aspects. Firstly, the purpose of this work is to reveal whether the geese show “head bobbing” phenomenon in head and neck movements; Secondly, the other purpose is to study the similarities and differences of head and neck movement characteristics of geese at different walking speeds.

The main object of this study was the gray Chinese goose (domestic goose). Through literature review, we familiarize ourselves with the physiological characteristics of the goose's head and neck, as well as the general movement characteristics and common movement indicators of the bird's neck. A high-speed camera system is used to capture video footage of the movement of the goose's head and neck in a straight-line walking state. By analyzing the captured video footage and processing the data, walking speeds and accelerations of domestic geese in a straight-line walking state are obtained under different walking conditions. The coordinates of head movements are measured, anchor points on the neck are marked, and movement data are collected. The movement cycles are observed, and gait analysis is combined with the analysis of head and neck movement characteristics. Comparisons are made with other bird species to determine the presence of head-bobbing behavior, and the laws of head and neck movement are summarized, with images depicting the movement characteristics and patterns being drawn.

## 2 Materials and methods

### 2.1 Experimental preparation

The main object of this study was the Chinese gray goose (domestic goose), a type of poultry domesticated from swans. We selected a 2-year-old Chinese domestic goose that weighed about 4 kg. Before the experiment begins, the goose is artificially reared by the experimenter for several days to familiarize it with the experimenters and the experimental site, ensuring the natural movement state of the subject. A perforated acrylic board with a specification of 80 × 80 cm is prepared as a background reference. The experiment used the Phantom Miro series M110 high-speed camera system (produced by Vision Research in New Jersey, USA) to capture video footage of geese walking in a straight line. The Labelme software (developed by MIT in Boston, USA) was used to process and store video clips. The computer used in the experiment was a Microsoft Surface laptop 2, equipped with a Windows 11 64-bit operating system, 8 GB of RAM and an Intel Core i5 8250 U processor. The computer is mainly used for running PCC software, storing and transcoding high-speed photographic images, and performing data processing.

High speed photography technology measurement data error mainly from the camera calibration process and the image processing is not accurate, because the PCC software saved images is usually slightly smaller than the original scene, in order to obtain the actual distance, angle, speed or acceleration parameters, we need to use the “calibration” function of PCC software to redefine the measurement ratio. After calibration, all measurements will be calculated and displayed in scale units at the time of calibration. In addition, the PCC software provides a “calculator” function to effectively improve the image quality to ensure that the location of the markers can be accurately extracted.

In addition, due to the random nature of a goose's natural walking behavior, it is difficult to consistently have it walk in a straight line at different speeds in front of the reference object in its natural state. To observe the movement characteristics of the research subject and facilitate later data processing, the following points should be considered in the preparation and experiment process:

1. A transparent acrylic plate was set up as the reference object, with holes drilled at specific locations to facilitate subsequent data measurement.
2. To reduce errors caused by the shooting angle, the camera was positioned perpendicular to the acrylic reference during the experiment.
3. To avoid flicker, daylight was used as much as possible, and additional lighting was only enabled when there was insufficient light.
4. The resolution, frame rate, exposure time, secondary exposure time, black balance, and other parameters of the high-speed camera were correctly set.
5. Artificially pull the goose of the experimental object to one side of the reference, and then drive it to the other side. In order to keep their walking gait more natural, the driving behavior is not easy to be too intense.

### 2.2 Experimental data collection method

The experiment uses an 80 × 80 cm perforated acrylic board as a reference object, with the high-speed camera set up directly facing the acrylic board. Parameters such as resolution, frame rate, exposure time, secondary exposure time, and black balance are adjusted accordingly. The shooting scene is inspected to ensure that the area is complete and that the subject goose, along with its neck marking points, are clearly visible without any obstructions.

After the camera operator starts recording, the goose is released to capture its complete straight-line walking process. Filming stops after the goose crosses the sampling area. The goose is then manually guided to one side of the reference object, and the filming process is repeated.

**Figure 1:**
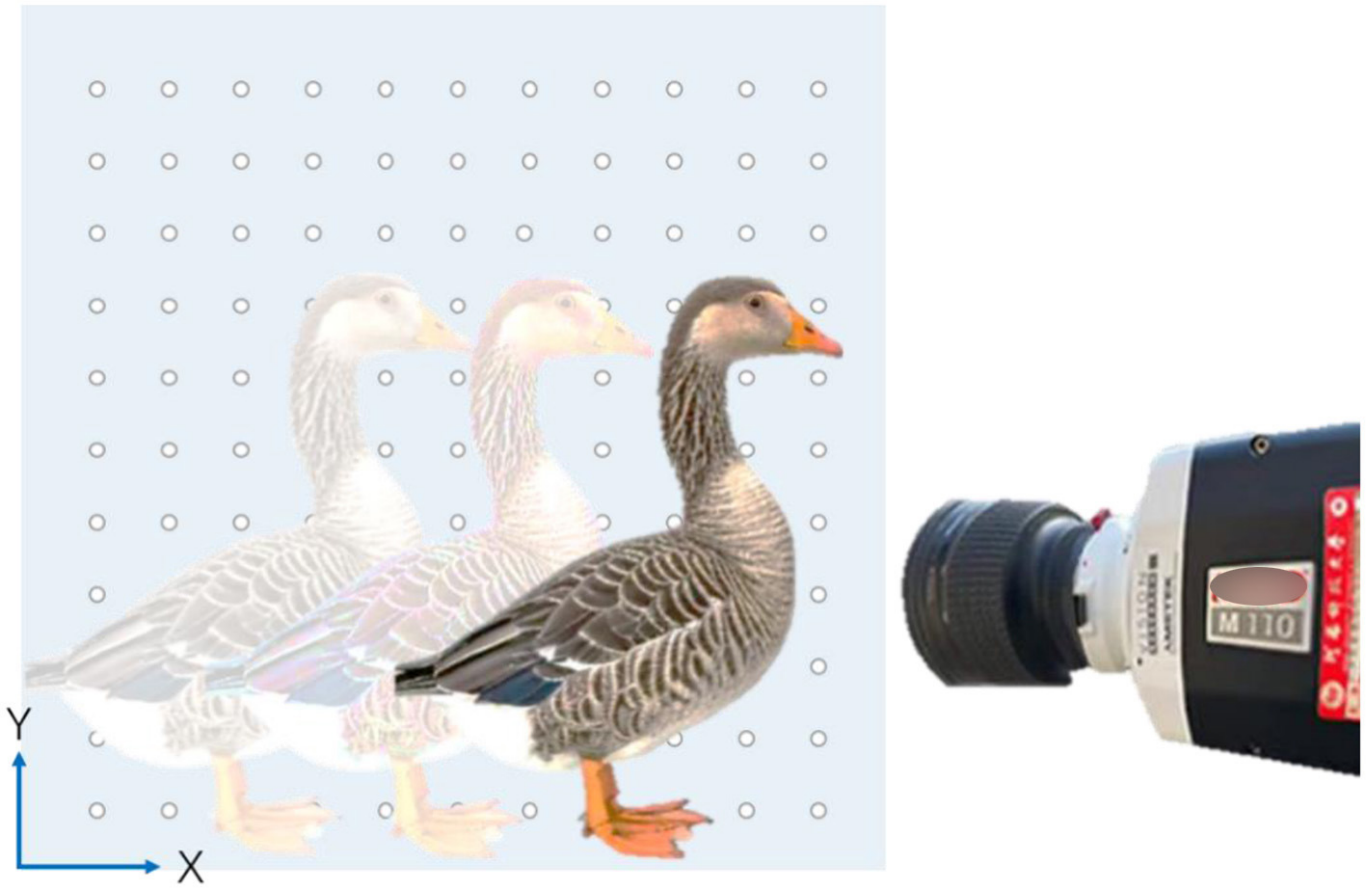
Experimental design.

After the filming is completed, the camera operator saves the video footage that meets the requirements. Six sets of footage are selected for analysis and are divided into three different speed groups based on walking speed: slow walking (A1, A2): 0.3 ≤ average speed ≤0.7 m/s, normal walking (B1, B2): 0.7 < average speed ≤0.9 m/s, and fast walking (C1, C2): average speed > 0.9 m/s, with two video data per group. Among them, three groups are designated as typical groups (numbered A2, B2, C2), and three groups as supplementary groups (numbered A1, B1, C1). The experimental process is shown in Figure 1.

The main imaging parameters of the RAW file format captured are shown in Table 1.

**Table 1:** Main imaging parameters.

**Parameter**	**Description**
Format	Raw cine file
Shutter mode	Global shutter
Camera model	Phantom Miro M110
Resolution	1,280 × 720
Sampling frequency (frame rate)	200 fps
Period	5,000.00 μs
Exposure time (μs)	4,999.540
Raw acquisition bit depth	12
Bits per color	P10 (Packed 10 log)

The motion video data was processed through PCC software, where videos corresponding to three walking patterns are selected, and their motion image sequences are extracted. The bottom left corner of the perforated acrylic board is set as the origin of the coordinate axis. Markers are created in the software, and based on the video's frame rate, the “step” between adjacent photos is set to 20 frames, meaning each step advances the video by 0.1 s. The software automatically measures the displacement, velocity, acceleration, and other information of the markers at the step interval as the minimum time unit.

**Figure 2:**
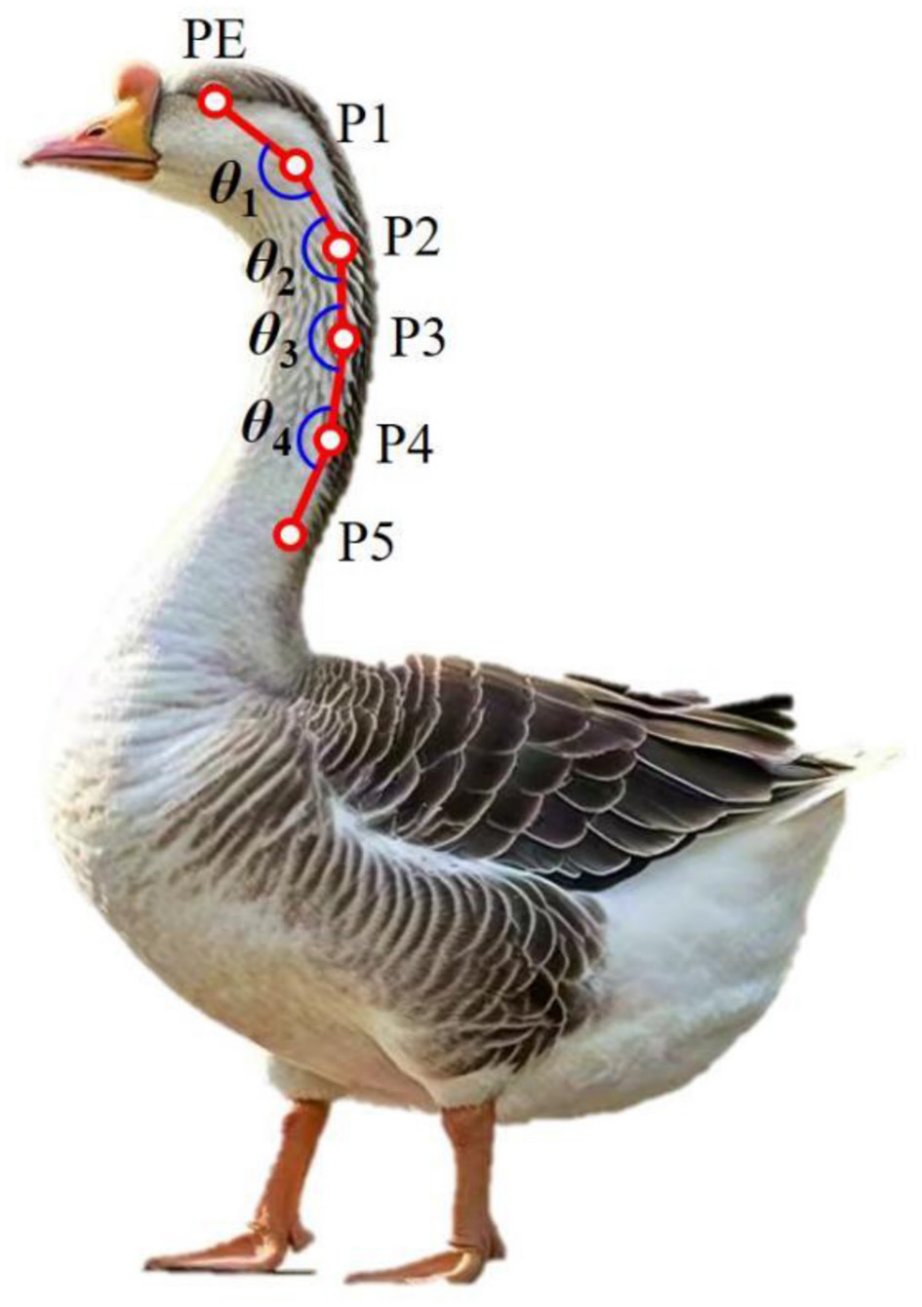
Marker point positions, PE represented the eye; P1, P2, P3, P4, and P5 represented the 2nd, 5th, 9th, 12th, and 15th cervical vertebra; θ_1_, θ_2_, θ_3_, and θ_4_ represented the angle between the lines connecting adjacent points.

The bone markers involved in this study were determined from the structural images of goose neck bone acquired using the biplane X-ray motion capture system (ISSI, Milpitas, CA, USA) used in literature (26). As showed in Figure 2, we used the red marker pen to establish marker points at the eye, 2nd, 5th, 9th, 12th and 15th cervical vertebra, marking PE, P1, P2, P3, P4 and P5, respectively. The change in the PE-P1-P2 angle was chosen to indicate the movement of the goose head. The change of the P1-P2-P3 angle indicates the movement of the upper cervical segment of the goose cervical spine, and the change of the P2-P3-P4 angle indicates the movement of the P3-P4-P5 angle indicates the movement of the middle goose cervical spine. They are labeled asθ_1_, θ_2_, θ_3_, and θ_4_, respectively. The angle measurement range is ±(0–180°). The joint angle symbol is defined as “+” when the cranial vertebra is reversed to the ventral side with respect to the caudal vertebra, and “–” when the cranial vertebra is reversed to the dorsal side with respect to the caudal vertebra.

## 3 Results

### 3.1 Walking speed and acceleration

**Figure 3:**
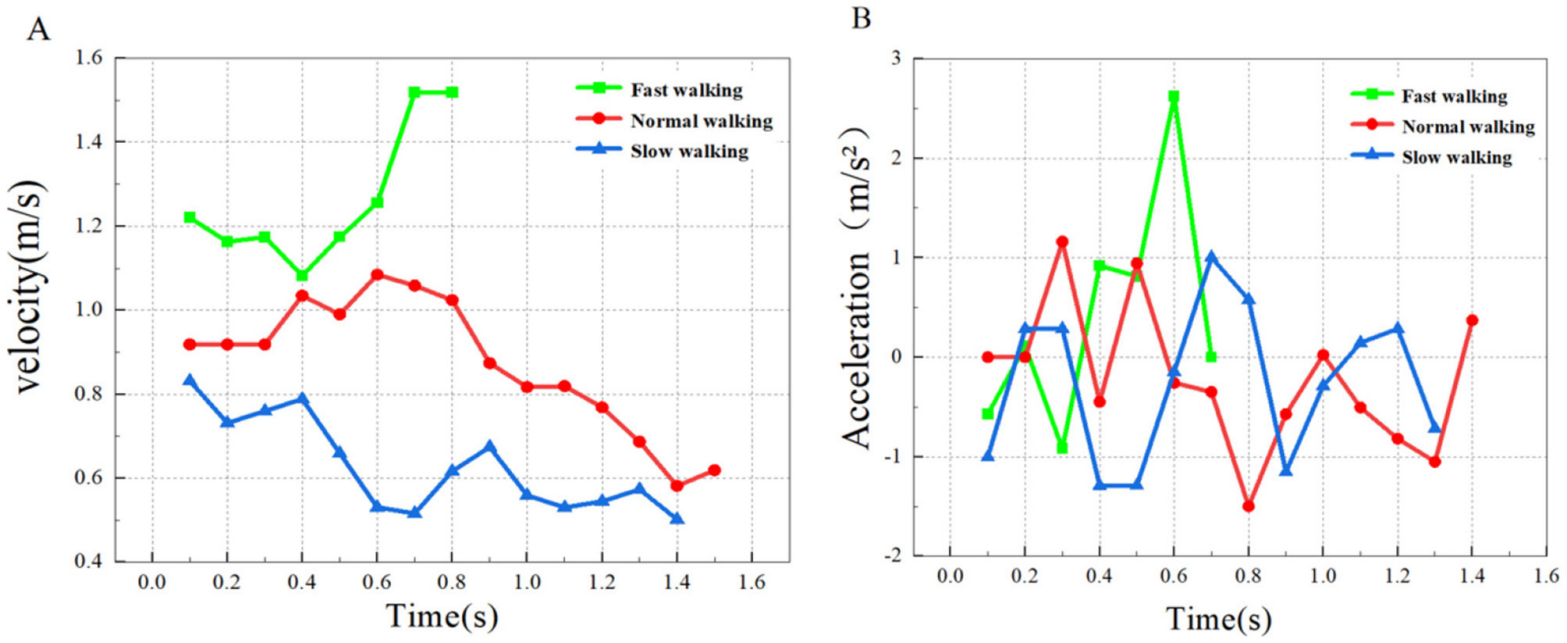
Walking speed and acceleration analysis. **(A)** The velocity change of the goose in a straight line; **(B)** Acceleration change of domestic goose in the straight walking state.

Motor data of the typical groups (slow, normal, and fast walking) measured in this experiment were extracted. According to the measurement data such as speed, time and distance, the acceleration and average speed of the three typical domestic goose movements can be calculated, and imported into the origin drawing software to draw images, as shown in Figure 3.

The data measured by the PCC software was saved as a Excel document, and Table 2 was created based on the speed and acceleration ranges measured by the six sets of data. As can be seen from Table 2, with the average velocity of the goose in the three straight walking states, the range of walking acceleration also shows a trend of gradual expansion. In addition, during fast walking (C2), goose acceleration was significantly higher than slow and normal walking. Meanwhile, the magnitude of the deceleration amplitude caused by the gait alternation was also significantly smaller than that in the previous two walking states. However, the range of speed did not show a single upward or downward trend with increasing walking speed.

**Table 2:** Range of velocity and acceleration variation.

**Walking states**	**Slow walking**		**Normal walking**		**Fast walking**	
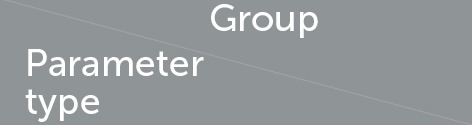	**A1**	**A2**	**B1**	**B2**	**C1**	**C2**
Range of speed variation (m/s)	0.325~0.545	0.502~0.832	0.407~1.068	0.582~1.084	0.746~1.148	1.082~1.518
Speed difference (m/s)	0.220	0.330	0.661	0.502	0.402	0.436
Range of acceleration variation (m/s^2^)	−1.150~0.831	−1.290~1.004	−1.626~0.348	−1.498~1.162	−1.162~1.779	−0.571~2.622
Acceleration difference (m/s^2^)	1.987	2.294	1.984	2.660	2.941	3.193
Average speed (m/s)	0.462	0.628	0.748	0.880	0.960	1.261

### 3.2 Range of head and neck motion

The experiment revealed that domestic geese exhibit different gaits at different walking speeds. To further clarify the motion characteristics of the goose's head and neck at different walking speeds, the data of goose head and neck joint at different walking speeds were measured and analyzed, and the results are shown in Table 3.

**Table 3:** Variation range of head and neck mobility in three walking states.

**Walking states**	**Slow walking**		**Normal walking**		**Fast walking**	
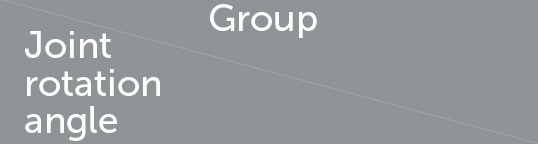	**A1**	**A2**	**B1**	**B2**	**C1**	**C2**
θ_1_	134.4°~151.3°	126.0°~144.1°	132.3°~150.1°	139.4°~158.1°	132.8°~155.1°	137.3°~154.9°
θ_2_	158.8°~167.0°	139.3°~158.3°	154.1°~168.1°	149.9°~169.4°	158.3°~168.0°	155.8°~165.8
θ_3_	163.0°~172.9°	157.2°~172.0°	160.7°~173.3°	168.2°~176.5°	158.6°~169.1°	163.5°~170.2°
θ_4_	166.2°~183.2°	165.3°~185.4°	169.0°~182.7°	166.0°~184.6°	173.7°~188.3°	175.1°~189.5°

From the data in Table 3, we can clearly observe that as the walking speed increases, the goose's head-lifting movement becomes increasingly evident, and the entire neck exhibits an increasing backward-bending trend with the increase in walking speed. In addition, during fast walking, the curvature of the lower part of the neck also increases.

**Figure 4:**
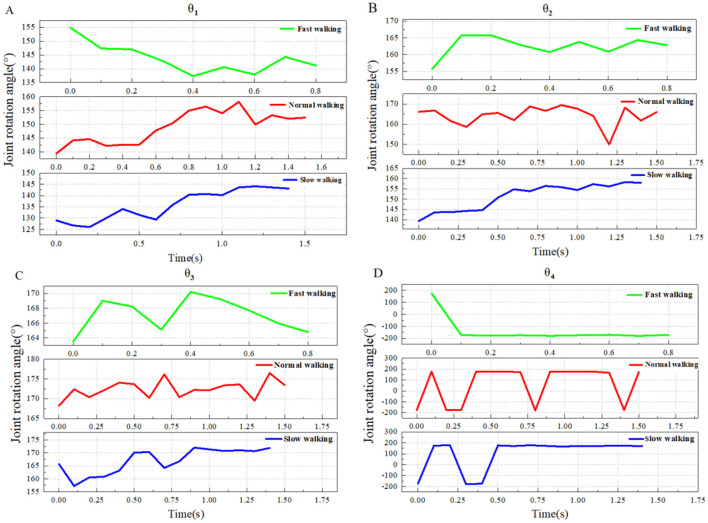
The transformation trends of head and neck joint angles (θ_1_-θ_4_) under three different motion states in a typical group. **(A)** Comparison of θ_1_ (PE-P1-P2) changes in three motion states; **(B)** Comparison of θ_2_ (P1-P2-P3) changes in three motion states; **(C)** Comparison of θ_3_ (P2-P3-P4) changes in three motion states; **(D)** Comparison of θ_4_ (P3-P4-P5) changes in three motion states. The joint angle symbol is defined as “+” when the cranial vertebra is reversed to the ventral side with respect to the caudal vertebra, and “–” when the cranial vertebra is reversed to the dorsal side with respect to the caudal vertebra.

Taking into account the data on goose head and neck movements at the three different walking speeds mentioned in the manuscript, the motion change curves of θ_1_- θ_4_ for the typical groups (numbered A2, B2, C2) are plotted, as shown in Figure 4.

From Figure 4, with the improvement of walking speed, the domestic goose head movement is gradually obvious, the whole neck shows a straight trend of increasing amplitude, and the reverse curvature amplitude of the lower part of the neck also increases gradually when walking fast.

**Figure 5:**
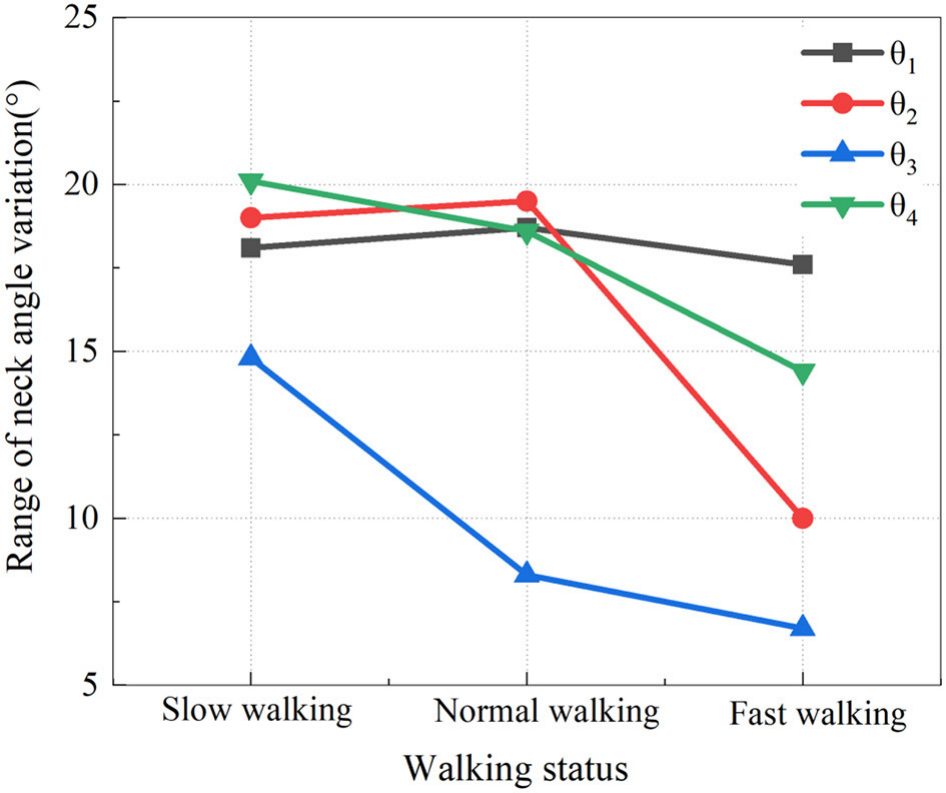
Relationship between the range of angle variation and walking state.

To more clearly reveal the relationship between the range of head and neck angle changes and walking states, we selected data from the typical groups (A2, B2, C2) and presented the range of their head and neck movement angles in graphical form. In Figure 5, the range of changes for θ_1_-θ_4_ is labeled respectively under increasing walking speeds.

From Figure 5, it can be seen that when accelerating from slow walking to normal walking, only θ_1_ and θ_2_ show a slight increase in the range of head and neck movement angles, with a minimal increase of just 1°, which is within the normal error range. In the three states of walking, the overall change of the head and neck range of motion has a downward trend. Specifically, the head and neck present smaller angles when the measurement point is closer to the head, but rather larger when the measurement point is closer to the trunk. A smaller axial angle is seen near the trunk and the joints near the head are larger.

### 3.3 Nodding behavior

**Figure 6:**
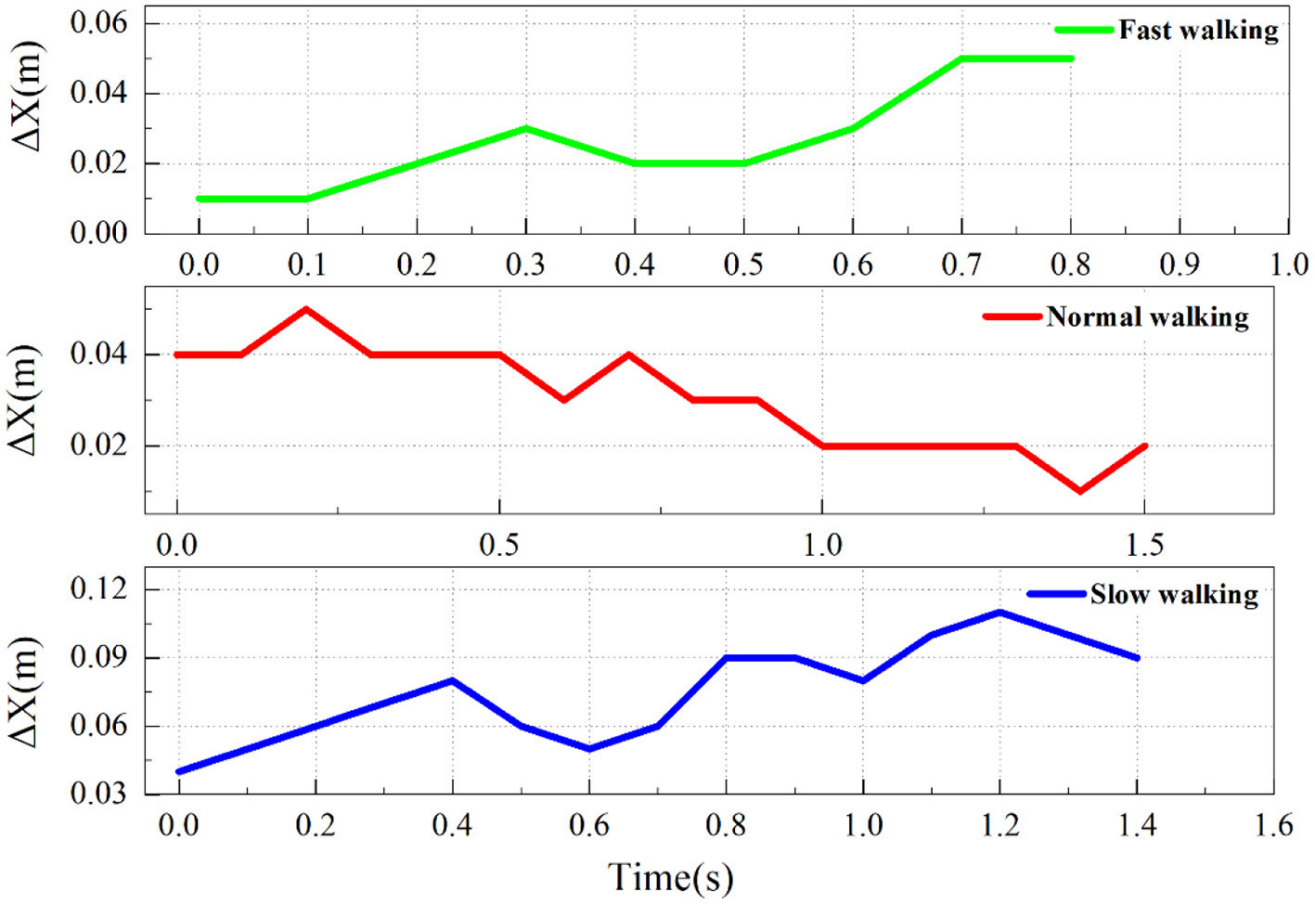
Nodding behavior under three movement states (Δ*x* is the coordinate difference between the marker point E and the P5).

In this experiment, through observation of high-speed photographic imagery, it was found that domestic geese exhibit nodding behavior similar to other birds during walking. To further investigate nodding behavior, we used the *x*-coordinate difference (Δ*x*) between markers E and 5 to represent the horizontal motion trajectory of the domestic goose head relative to the neck. After the initial measurements, the motion characteristics of the three states were plotted, as shown in Figure 6.

**Figure 7:**
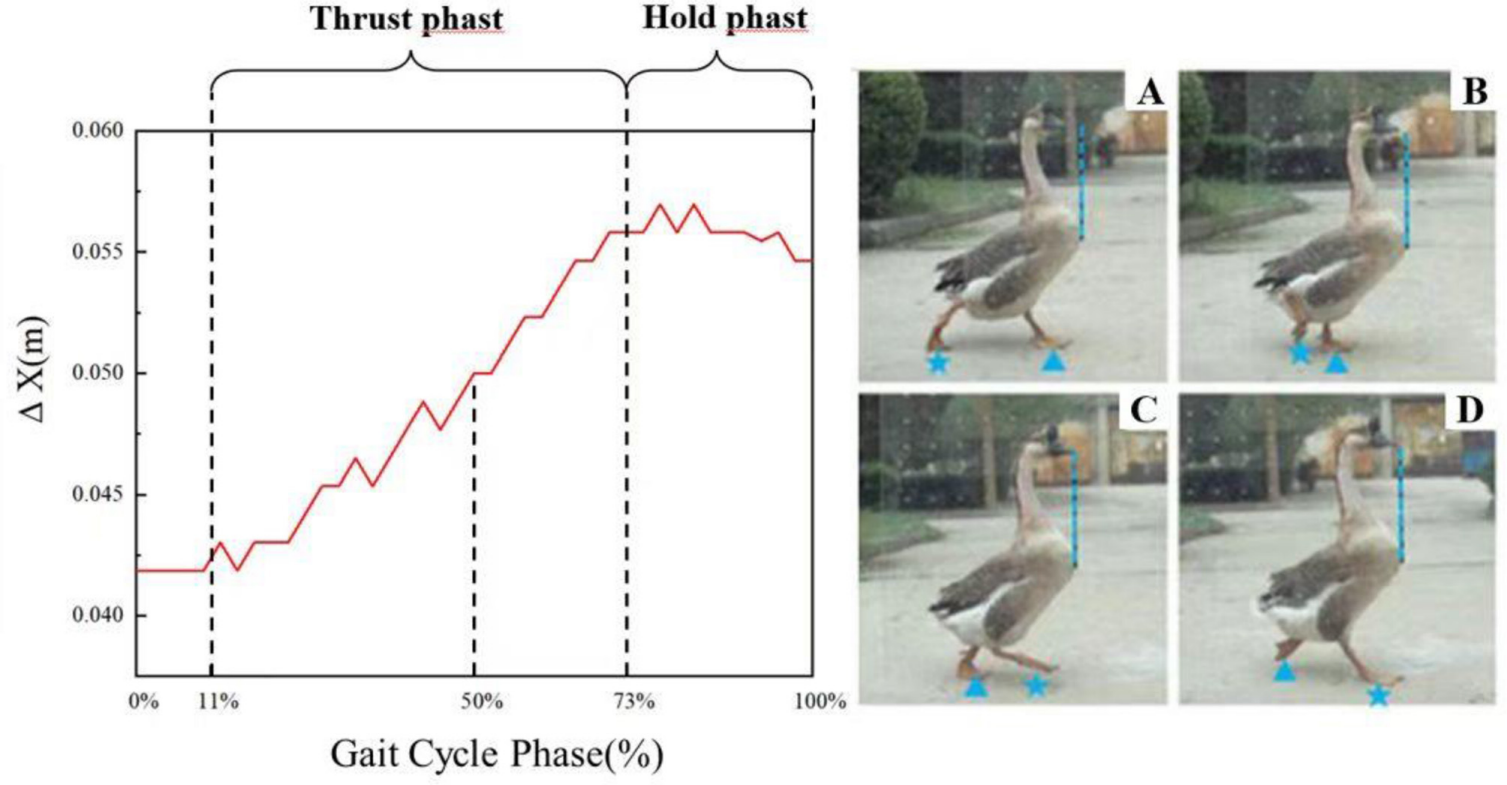
The standardized diagram of head bobbing movement during half a gait cycle. **(A)** the initiation of the rear foot; **(B)** the image during thrust phase; **(C)** the end of the thrust phase and the start of the hold phase; **(D)** the landing of the rear foot. The vertical blue dashed lines mark the target positions for the goose's head to reach within half a gait cycle. The foot with a blue triangle marker is defined as the front foot, and the foot with a blue star marker is defined as the rear foot.

By comparing and analyzing the high-speed photographic images, it was preliminary confirmed that the domestic goose showed a nodding behavior during walking. Results show that geese exhibit more obvious nodding movements when fast walking compared to normal and slow walking. To better illustrate the nodding behavior, we selected typical data from group C2 during quick walking and further analyzed the goose nodding movement characteristics during the gait cycle, as shown in Figure 7.

Figure 7 showed the standardized diagram of head bobbing movement during half a gait cycle. Four images during the gait cycle were also showed and numbered from A to D. The nodding movement of goose is divided into a thrust phase and a hold phase. The stage from 11 to 73% of the half a gait cycle represents the thrust phase, while the stage from 73% to next 11% of the next half a gait cycle represents the hold phase. As shown in Figures 7A–D, the changes in head and neck posture (indicated by the blue dashed line) and feet positions (indicated by blue triangles and star shapes) of a goose during linear movement are depicted.

A complete gait cycle of the goose starts with each one foot leaving the ground and ends with the other foot landing. A half of gait cycle starts with the rear foot leaving the ground and ends with the same foot landing. Take the right foot as an example, Figure 7 showed half a gait cycle. As we all known, the goose behaves the same nodding movements regardless of which foot steps forward. Therefore, we take the half-gait cycle as an example to analyze the nodding motion during gait cycle. Figure 7A shows the starting position of the gait cycle, which is not the starting point of the thrust phase of the head bobbing motion. When the goose moves from the starting position in Figures 7A, B, the rear foot moves forward, however, the goose's head moves forward relative to its neck from the 11% of the half a gait cycle. During the transition from Figures 7B, C, the head moving forward while the rear foot continues forward. Throughout the movement, the goose's head extends forward relative to the body. At this point, the thrust phase ends and the position represents 73% of half a gait cycle. When the goose reaches from Figures 7C, D, the head remains stationary relative to the neck, at the time point of Figure 7D, the head returns to its normal position relative to the neck and the rear foot touches the ground. Subsequently, the left foot becomes the rear foot, the next half a gait cycle starts. Noticeably, the hold phase starts from Figure 7C, and it continues for a short period of time after the rear foot takes a step forward in the next half gait cycle.

As shown in Figure 7, the thrust phase, accounting for 62% of the half a gait cycle. During this phase, the head is always extended relative to the neck as the goose steps forward. The stage from 73% to next 11% of the half a gait cycle represents the thrust phase, where the head moves backwards relative to the neck and makes a nodding motion.

**Figure 8:**
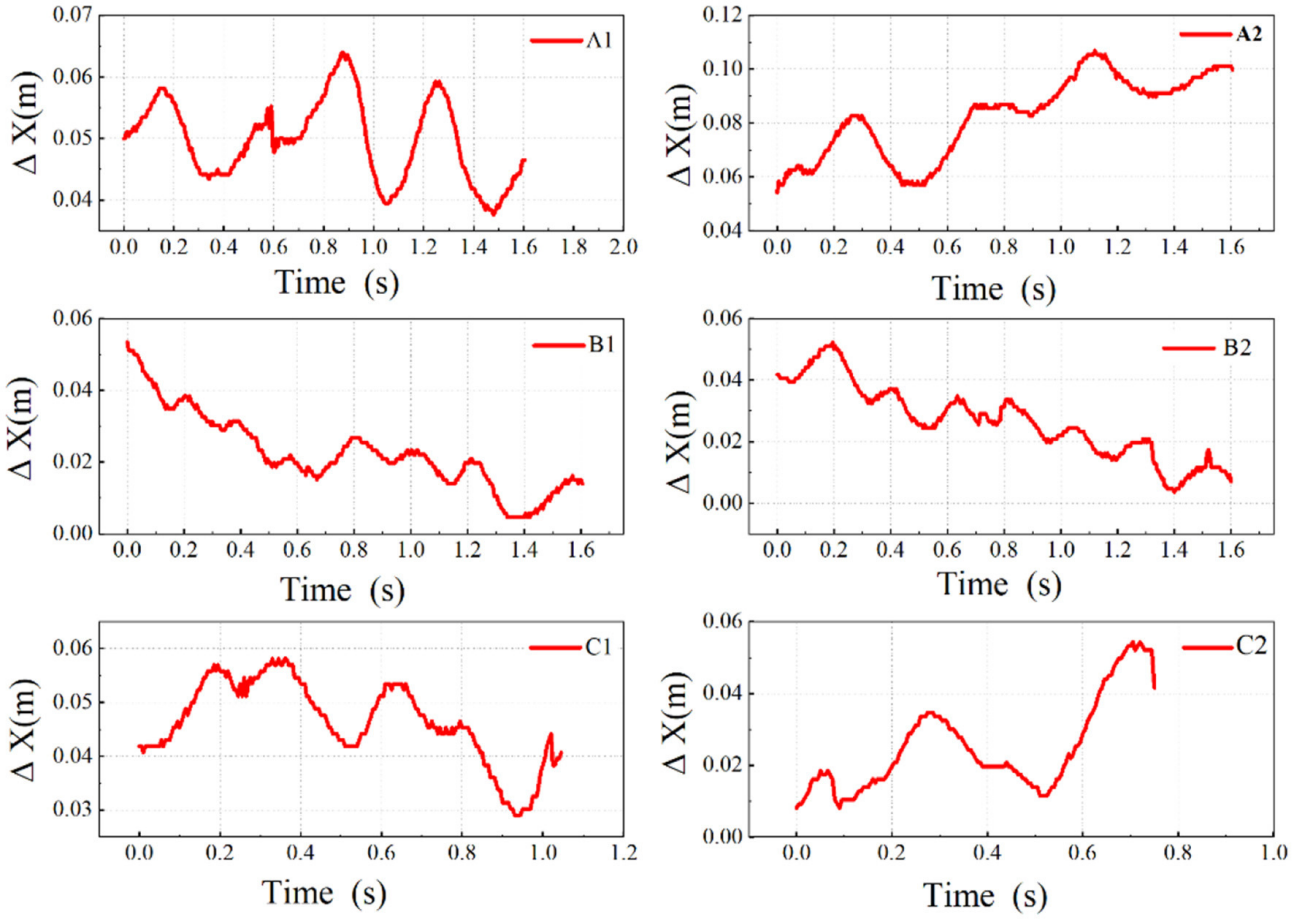
Comparative analysis of nodding movement in different movement states. Δ*x*-coordinate difference between eye and P5; A1, A2-slow walking; B1, B2-normal walking; C1, C2-fast walking.

It was preliminary confirmed that domestic geese could show similar nodding behavior as other birds during exercise, however, this characteristic is not as pronounced in the geese as it is in other short-necked species. To further confirm this phenomenon, we improved the accuracy of the sampling points and reduced the sampling interval for further analysis. Images plotted for all six sets of data are shown in Figure 8.

By increasing the sampling frequency, reducing the sampling interval, and analyzing frame by frame, the curves reveal that domestic geese indeed exhibit nodding behavior that coordinates with their steps during walking. Notably, in Figure 8A2, two distinct nodding actions followed by sustained postures can be observed at 0.43–0.51 s and 0.68–0.82 s. Similar phenomena are also present in Figures 8A1, C1, indicating that the nodding curves of domestic geese during walking are not always continuous fluctuations as previously estimated, but rather exhibit propulsion and hold phases in some instances. Similarly, the durations of the thrust phases preceding the two holding phases in A2 were measured again, lasting from 0.29 to 0.42 s and 0.52 to 0.67 s, respectively. The proportions of the propulsion and holding phases in the two nodding actions were 60.9% and 39.1%, and 51.7% and 48.3%, respectively. Additionally, in A1, the proportions of the thrust and hold phases were 61.3 and 38.7%, while in C1, they were 63.6% and 36.4%. In a complete step, the thrust phase is always longer than the hold phase. This thrust and hold pattern can be observed during both slow and fast walking but is not evident during normal walking.

Meanwhile, during the retraction phase of the nodding action, the head moves backward relative to the neck but continues to move forward relative to the environment. This suggests that nodding is more of a special movement pattern where the head periodically moves ahead of the neck during motion.

### 3.4 Head movement trajectory

**Figure 9:**
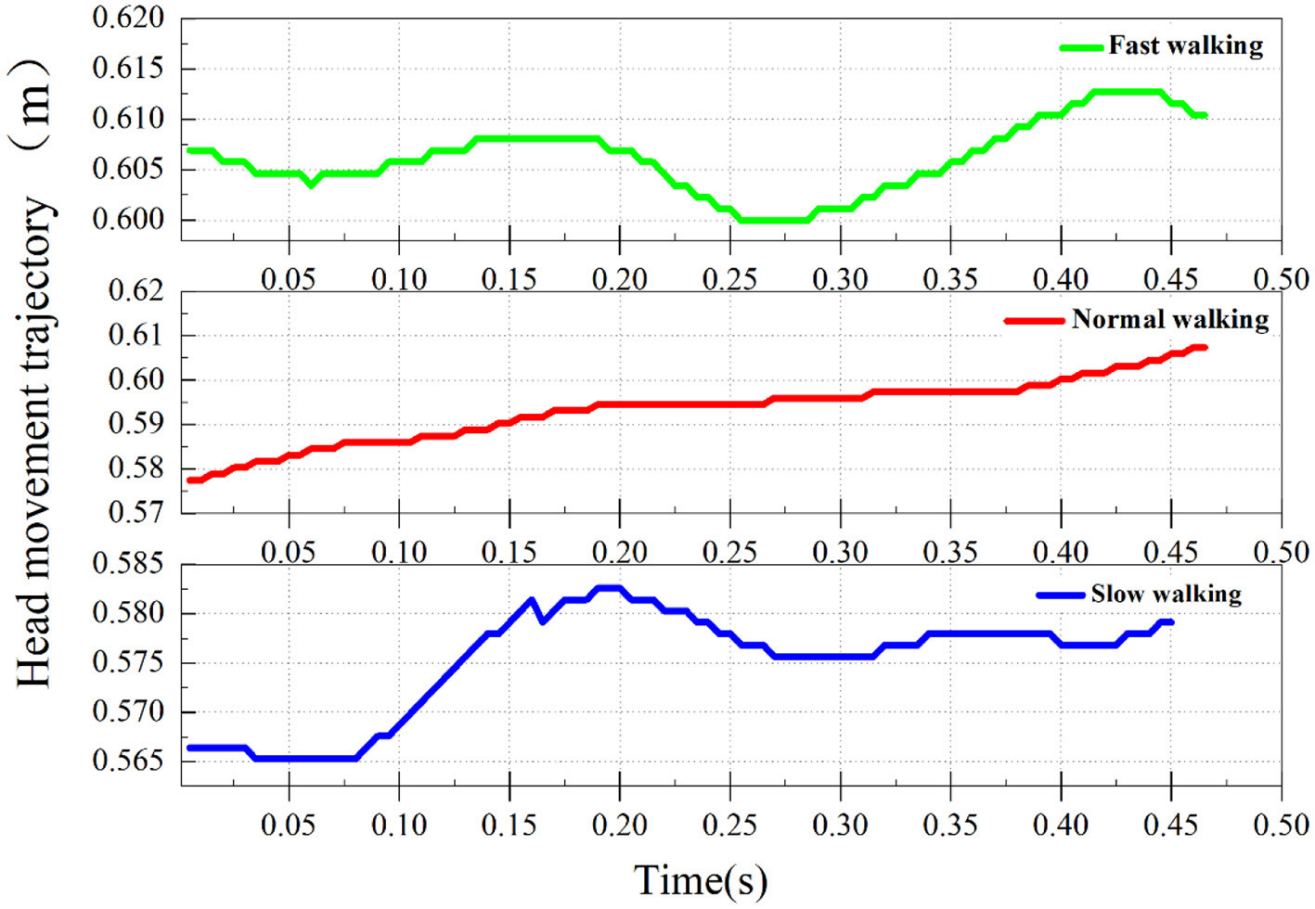
The straight-line walking head trajectory of a domestic goose.

After finding the nodding movement of the goose during straight line walking, the head movement trajectory of the goose during straight line walking was measured. The eye position data of the complete gait cycle of domestic geese at different walking speeds were measured and sorted, and the head movement trajectory of three sets of data from the typical group was plotted and shown in Figure 9.

From Figure 9, it can be seen that the head movement trajectories of geese exhibit similarity when they walk in a straight line at different speeds. During each full gait cycle, the goose head makes a slight nodding movements in the *Y* axis direction. This pattern persists during fast walking, but the second step trajectory becomes less noticeable due to the reduced amplitude of head movement.

In addition, in the typical slow-walking group A2 (the group with a noticeable hold phase in the nodding analysis), apart from the hold phase in the *X*-axis direction, a holding phenomenon in the *Y*-axis direction was also observed. This phenomenon occurs at the highest point of the trajectory on the *Y*-axis and is also present in the other two groups.

Therefore, we further measured the specific changes in the *Y*-axis coordinates, with the results shown in Table 4. In the *Y*-axis direction, the head fluctuation ranges for the typical groups (A1, B1, C1) in the three straight-line walking states are: 0.577–0.623 m (Δ*Y* = 0.046 m) for slow walking, 0.600–0.613 m (Δ*Y* = 0.013 m) for normal walking, and 0.573–0.583 m (Δ*Y* = 0.01 m) for fast walking. With the increase in walking speed, the fluctuation ranges decreased by 72% and 23%, respectively. Among the six sets of data with increasing walking speeds, the amplitude of head up-and-down movement showed a decreasing trend. In all six sets of data, the amplitude of head up-and-down movement exhibited a decreasing trend with the increase in walking speed.

**Table 4:** Range of head fluctuations in the three walking states.

**Walking states**	**Slow walking**		**Normal walking**		**Fast walking**	
**Group**	**A1**	**A2**	**B1**	**B2**	**C1**	**C2**
*Y*/*m*	0.583–0.62	0.577–0.623	0.573–0.579	0.600–0.613	0.572–0.586	0.573–0.583
Δ*Y*/*m*	0.037	0.046	0.021	0.013	0.014	0.010

## 4 Discussion

This paper investigates the head and neck movement patterns of domestic geese during straight-line walking, we demonstrate that domestic geese will show similar nodding behavior as other birds at different walking speeds. Previous studies have shown that nodding motion is crucial for maintaining visual stability in birds during walking and flying. For example, by analyzing the movements of the head and body when the pigeons make 90° turns, Ros and Biewener (28) proved that the line of sight can adjust by moving the head to obtain depth of field information, which helps the pigeons maintain balance during movement and avoid collision. However, unlike other small birds (pigeons, quail) that rely on the upper cervical spine, goose nodding behavior is mainly driven by the lower cervical spine (θ_4_ is the most variable compared to other angles). During the walking of the goose, the reverse movement of the lower segment of the neck is coordinated with the advancing and holding stages of the head, helping the goose to maintain the relative stability of the head during the step. This coordination mechanism allows the goose to maintain the balance of the head through fine tuning of the neck during walking, especially during fast walking, where the amplitude of the reverse curvature of the lower neck segment increases, further enhancing the head stability.

In addition, this paper confirms that the domestic goose's head will show a certain degree of “hold phase” in the vertical direction during the movement. With the increase of the movement speed, the head fluctuation amplitude of the domestic goose showed a downward trend. This indicates that the domestic goose can achieve relative head stability in the vertical direction by adjusting the frequency and amplitude of the nodding movements. This behavior of keeping the head stable in the vertical direction is similar to that of other birds. For example, Nyakatura and Andrada (6) showed that quail achieve a dynamic equilibrium mechanism during walking. This mechanism not only helps quail to reduce energy expenditure during rapid movement, but also uses nodding behavior to effectively offset the vertical shaking caused by pace.

The limitations of this study including the following two aspects: Firstly, this paper only studied the head and neck movement characteristics of domestic geese during straight-line walking in a two-dimensional plane from the side, without exploring left-right swaying by designing experiments facing the geese's direction of travel. Further research should be conducted in a plane perpendicular to the direction of travel. Secondly, this paper only studied the head and neck movement characteristics of domestic geese at three different walking speeds, without dividing the gait into multiple categories such as walk, amble, pace, trot, and gallop as in studies of quadrupeds. Future research should refine the definition of bipedal gait and explore movement characteristics under more conditions.

In conclusion, by studying the pattern of head and neck movements in domestic geese during straight walking, we not only revealed the driving mechanism of domestic goose nodding behavior, but also further explored the coordinated relationship between nodding movements and gait and its influence on head stability. These results not only provide a deeper understanding of visual stability and balance control mechanisms during bird movement, but also provide useful references and inspirations for fields such as bionics and robotics.

## Data Availability

The original contributions presented in the study are included in the article, further inquiries can be directed to the corresponding authors.
